# Comparing ATPase activity of ATP-binding cassette subfamily C member 4, lamprey CFTR, and human CFTR using an antimony-phosphomolybdate assay

**DOI:** 10.3389/fphar.2024.1363456

**Published:** 2024-02-19

**Authors:** Guiying Cui, Kerry M. Strickland, Analia J. Vazquez Cegla, Nael A. McCarty

**Affiliations:** Division of Pulmonology, Asthma, Cystic Fibrosis, and Sleep, Department of Pediatrics, Emory + Children’s Center for Cystic Fibrosis and Airways Disease Research, Emory University School of Medicine and Children’s Healthcare of Atlanta, Atlanta, GA, United States

**Keywords:** ATPase activity, human ABCC4, human CFTR, lamprey CFTR, Sf9 cell, baculovirus

## Abstract

**Introduction:** ATP-binding cassette (ABC) transporters use the hydrolysis of ATP to power the active transport of molecules, but paradoxically the cystic fibrosis transmembrane regulator (CFTR, ABCC7) forms an ion channel. We previously showed that ATP-binding cassette subfamily C member 4 (ABCC4) is the closest mammalian paralog to CFTR, compared to other ABC transporters. In addition, Lamprey CFTR (Lp-CFTR) is the oldest known CFTR ortholog and has unique structural and functional features compared to human CFTR (hCFTR). The availability of these evolutionarily distant orthologs gives us the opportunity to study the changes in ATPase activity that may be related to their disparate functions.

**Methods:** We utilized the baculovirus expression system with *Sf*9 insect cells and made use of the highly sensitive antimony-phosphomolybdate assay for testing the ATPase activity of human ABCC4 (hABCC4), Lp-CFTR, and hCFTR under similar experimental conditions. This assay measures the production of inorganic phosphate (P_i_) in the nanomolar range.

**Results:** Crude plasma membranes were purified, and protein concentration, determined semi-quantitatively, of hABCC4, Lp-CFTR, and hCFTR ranged from 0.01 to 0.36 μg/μL. No significant difference in expression level was found although hABCC4 trended toward the highest level. hABCC4 was activated by ATP with the equilibrium constant (K_d_) 0.55 ± 0.28 mM (*n* = 8). Estimated maximum ATPase rate (V_max_) for hABCC4 was about 0.2 nmol/μg/min when the protein was activated with 1 mM ATP at 37°C (*n* = 7). Estimated maximum ATPase rate for PKA-phosphorylated Lp-CFTR reached about half of hCFTR levels in the same conditions. V_max_ for both Lp-CFTR and hCFTR were significantly increased in high PKA conditions compared to low PKA conditions. Maximum intrinsic ATPase rate of hABCC4 in the absence of substrate was twice that of hCFTR when activated in 1 mM ATP.

**Conclusion:** The findings here suggest that while both ABCC4 and hCFTR bear one consensus and one degenerate ATPase site, the hCFTR exhibited a reduced intrinsic ATPase activity. In addition, ATPase activity in the CFTR lineage increased from Lp-CFTR to hCFTR. Finally, the studies pave the way to purify hABCC4, Lp-CFTR, and hCFTR from Sf9 cells for their structural investigation, including by cryo-EM, and for studies of evolution in the ABC transporter superfamily.

## 1 Introduction

The adenosine triphosphate (ATP) binding cassette (ABC) transporters represent a large superfamily of membrane proteins, expressed in both prokaryotes and eukaryotes. Many members in the ABC transporter family, including hABCC4/MRP4 (Multidrug resistance-associated protein, MRP4) and hCFTR, are clinically relevant since they are involved in disease development and treatment (Vasiliou et al., 2009; Ford and Beis, 2019; Habermann et al., 2020; Bickers et al., 2021). Unlike hCFTR which requires PKA/PKC phosphorylation and ATP binding to nucleotide binding domains (NBDs) to activate the channel, the movement of bound substrates across the membrane in hABCC4 is powered by the cooperative binding of ATP alone at the two sites in the interface of two NBD_S_. Like hCFTR, one of the two sites in hABCC4 is a degenerate site, incapable of ATPase activity, and the other is a consensus site (Bloch et al., 2023; Huang et al., 2023).

We previously reported hABCC4 is the closest mammalian paralog to hCFTR among the ABC transporter superfamily (Jordan et al., 2008). The presence of hABCC4 transcript and protein was further verified in human lung tissue and human airway epithelial cells by Kim and colleagues (Kim et al., 2021). In addition, hABCC4 is involved in complex roles as a transporter (Wu et al., 2005; Wittgen et al., 2012; Tanaka et al., 2014) and exhibits functional and physical associations with CFTR (Li et al., 2007). The Gaggar and McCarty laboratories recently led an effort to clone and characterize CFTR from the sea lamprey (Lp-CFTR) (Cui et al., 2019). Being the evolutionarily oldest CFTR ortholog cloned thus far, Lp-CFTR shares ∼46% sequence identity and ∼65% sequence similarity with hCFTR. Accordingly, Lp-CFTR differs from hCFTR in multiple functional characteristics, such as single channel behavior, ATP sensitivity, and pharmacological modulation (Cui et al., 2019; Infield et al., 2021).

It is well known now that lipids play a critical role not only in the structure but also the function of membrane proteins, including CFTR and ABCC4 (Hildebrandt et al., 2014; Abu-Arish et al., 2015; Hildebrandt et al., 2017; Abu-Arish et al., 2019; Dunn et al., 2020; Cui et al., 2021; Sun et al., 2021; Bloch et al., 2023). This notion has been further supported by native mass spectrometry, which additionally revealed that oligomeric states of membrane proteins are stabilized by the presence of specific endogenous lipids (Gupta et al., 2018). To rule out the potential effects of the lipid variability between expression systems on ATPase activity of the three target proteins, we utilized Baculovirus/Sf9 heterologous expression system to express hABCC4, Lp-CFTR, and hCFTR, and studied their ATPase activity using the Antimony-phosphomolybdate assay in the same lipid environment condition (Bartolommei et al., 2013; Bartolommei and Tadini-Buoninsegni, 2016).

## 2 Materials and methods

### 2.1 Bac-to-Bac baculovirus expression system and Sf9 cells for protein expression

Bac-to-Bac baculovirus expression system and Sf9 cells adapted to Sf-900 II SFM media (Cat #, 11496015, Cat # 10902104) used in this project were from ThermoFisher Scientific. Sf9 cell expansion, culture, and subsequent baculovirus experiments were performed following the company’s manual with slight modification (O'Shaughnessy and Doyle, 2011; Date et al., 2017). Three constructs were used in this project: human ABCC4 (hABCC4), Lamprey-CFTR (Lp-CFTR), and human CFTR (hCFTR), which were subcloned into the pFastBac Vector that contains a 6xhis tag by the Emory Integrated Genomics Core at Emory University. The three constructs were transformed into DH10Bac™ *E. coli* (ThermoFisher Scientific, Cat #, 10361012) and the bacmid DNA of the three constructs were purified using a ZR BAC DNA miniprep Kit (ZYMO RESEARCH, D4048). The bacmid DNAs were verified by PCR and full sequencing of the inserts. Sf9 cells were then chemically transfected with each individual BAC DNA using Cellfectin II reagent (ThermoFisher Scientific, Cat #, 10362100). The P0 viruses of the three BAC DNAs were collected and further amplified using Sf9 cells. P1 viruses of the three constructs were then collected, aliquoted, and stored at −80°C. P1 viruses of hABCC4, Lp-CFTR, and hCFTR were further used to infect Sf9 cells for protein expression.

### 2.2 Membrane preparation from Sf9 cells

Non-infected Sf9 cells and infected Sf9 cells were harvested by centrifugation at 1000xg for 10 min, washed with 5 mL of 1 X PBS, and pelleted at 1000xg for 10 min. The pellets were promptly frozen with liquid nitrogen and stored at −80°C. The cell pellets were resuspended in Buffer 1 (50 mM Tris-HCl, 250 mM sucrose, 0.25 mM CaCl_2_, pH 7.4) mixed with 1X protease inhibitors (Sigma P8340-1 mL, 1: 100 dilutions for final use) right before experiments (Hardy et al., 2019a; Hardy et al., 2019b). Cells were lysed using an ice-cold Dounce Homogenizer, 30 strokes with the loose pestle and then 30 strokes with the tight pestle on ice. The cell lysates were centrifuged at 750 *g* for 10 min at 4°C to remove cell debris; the supernatant was then ultracentrifuged at 37,500 rpm for 1 h at 4°C (Optima XPN-100 Ultracentrifuge, BECKMAN COULTER). The membrane pellets were resuspended in ice cold Buffer 2 (50 mM Tris-HCl, pH 7.4, 250 mM sucrose), aliquoted, and stored at −80°C. Proteins of hABCC4, Lp-CFTR, and hCFTR were evaluated with Western blotting and verified with anti-His Rabbit polyclone antibody (Cell Signaling, Cat #, 2365).

### 2.3 Semi-quantification of protein expression levels using immunoblotting and image densitometry

The total protein concentrations of membranes, including from controls (untransfected Sf9 cells), and Sf9 cells expressing hABCC4, Lp-CFTR, and hCFTR, were measured using a bicinchoninic acid (BCA) assay kit (ThermoFisher Scientific, Cat #23225). Specified amounts of individual Sf9 membranes containing target proteins and different amounts of the purified rat His-tag protein (PRHP) (Rat-Tie-2-His-tag protein, R&D, Cat # 10458T2050) were prepared in dithiothreitol-containing Laemmli sample buffer to a total of 42 µL each and incubated at room temperature for 15 min before loading in a gel (4%–15% Mini-PROTEAN TGXTM Precast Protein Gels, Bio-Rad, Cat # 4561084). The loaded samples were electrophoresed in Tris-glycine running buffer (25 mM tris base, 250 mM glycine, and 0.1% SDS) at 100 V for 60–70 min until the dye front reached the end of the gel. Proteins from the gel were transferred to a polyvinylidene fluoride (PVDF) membrane overnight at 4°C. The membrane was blocked with Intercept Blocking Buffer (*LI-COR*, Cat # 927-70001) for 1 h at room temperature. Blots were probed with a rabbit anti-His poly-clone antiserum at a dilution of 1:500 (Cell signaling, Cat# 2365), followed by IRDye 800CW Goat anti-Rabbit IgG secondary antibody at a dilution of 1:10,000 (*LI-COR*, Cat # 926-32211). The blots were visualized using Odyssey imager and software from *LI-COR*.

The western blotting images were further analyzed using ImageJ (Janes, 2015; McDonough et al., 2015; Kendrick et al., 2020). Band density ratios were calculated relative to the highest PRHP load. The absorbance values for each image were background subtracted, plotted as band density *versus* load and fit with a hyperbolic equation (Single Rectangular, 2 parameter) in Sigmaplot 12.3 (Palo Alto, CA). The regression line has an adjusted correlation *R* ^2^ value of 0.97–0.999 (Tang et al., 2023). The protein concentrations of hABCC4, Lp-CFTR, and hCFTR were calculated following the fit result of the standard PRHP load in the same image. Absorbance values were always within the linear range of the standard curves for PRHP abundance.

### 2.4 Antimony-phosphomolybdate assay/method for ATPase activity measurement

We adopted the Antimony-phosphomolybdate method developed to detect small amounts of inorganic phosphate (P_i_) to evaluate the rate of hydrolysis of ATP by phosphatases/ATPase (Bartolommei et al., 2013; Bartolommei and Tadini-Buoninsegni, 2016). The experimental procedures were carried out at room temperature unless otherwise stated. Each procedure consists of the preparation of a coloring solution, the determination of a calibration curve with a series of standard solutions of P_i_, and the spectrophotometric quantification of P_i_ released by the ATPase protein at subsequent time intervals. Coloring solution is made fresh for every experiment from stock solutions to reach final concentrations of 125 mM H_2_SO_4_, 0.5 mM ammonium molybdate, 10 mM ascorbic acid, 40 µM tartrate, and double distilled H_2_O (ddH_2_O) at room temperature. The stock solutions are made and stored individually, including 2.5 M H_2_SO_4_ (Sigma, 339741), 24 mM Ammonium molybdate solution (Sigma, 1.01180), 0.3 M ascorbic acid (Fisher Scientific, BP351-500), and 4 mM potassium antimony (III) oxide tartrate trihydrate (Sigma, 1.08092). Standard phosphate solutions (KH_2_PO_4_, Sigma, P0662) are made by serial dilution of 1 mM phosphate stock in ddH_2_O to get final solution of P_i_ from 0 to 100 nM. Thirteen standard solutions were prepared at 100 µL each to which 900 µL of coloring solution was added (final volume of 1,000 µL per tube). Color development starts immediately, stops when it plateaus and remains stable for hours. 200 μL of 1,000 µL of every standard solution was added to a 96 well transparent plate (BRANDplates, Cat# 781601) and the color was detected with absorbance at 850 nm (SpectraMax iD3 driven with SoftMax Pro7, Molecular Devices). The calibration curve was obtained by plotting the absorbance *versus* P_i_ concentration and fitted with the Polynomial, Linear equation in SigmaPlot 12.3.

The ATPase activity measurement was performed using the crude membranes purified from control Sf9 cells and Sf9 cells expressing hABCC4, Lp-CFTR, or hCFTR based on the study by Bartolommei and colleagues with modification (Bartolommei et al., 2013; Bartolommei and Tadini-Buoninsegni, 2016). The following solutions were mixed sequentially: Buffer 2, protein kinase A (2,500 units/µL) (PKA catalytic subunit, New England Biolabs, Cat #P6000), MgCl_2_, various concentrations of Na_2_ATP (Sigma, Cat# A6419), and finally the membrane samples to the total volume of 100 µL for every measurement in room temperature. No PKA was added for ATPase activity measurement of hABCC4. The volume of Buffer 2 was adjusted to retain final volume. The final concentration of MgCl_2_ is 5 mM for all the measurement (Sigma, M2670). For Sf9 membranes bearing Lp-CFTR and hCFTR, the tubes were incubated at 30°C for 1 h, then 900 µL fresh color solution was added to each tube to stop the reaction and incubated at room temperature for 2 h for color development. For control only and membranes bearing hABCC4, solutions were mixed sequentially to a final volume of 100 µL for each tube at room temperature and then were incubated at 37°C for 20 min instead. Then 900 µL of fresh color media was added to each tube and these were then maintained at room temperature for 2 h for hABCC4. A final volume of 200 µL of every sample was added to the same 96 well transparent plate with their standard solutions and the plate was read with absorbance at 850 nm. This test condition for hABCC4 (20 min incubation at 37°C) was widely used by previous publication (Sauna et al., 2004; Huang et al., 2023). The test conditions for hCFTR/Lp-CFTR (1 h at 30°C) were selected to enable the best efficacy of PKA (NEB, Cat #P6000) (Zhang and Chen, 2016; Liu et al., 2017). We used these experimental conditions in most of this project with the exception of the final figure for direct comparison of hABCC4 and hCFTR (1 h, 30°C incubation), as listed in the results.

### 2.5 Statistical analysis

Results are presented as mean ± SEM. Data was analyzed using Sigmaplot 12.3. Student’s t-test were performed for single comparisons. A *p*-value <0.05 was accepted to be a statistically significant difference between groups.

## 3 Results

### 3.1 Optimization of the experimental conditions for color development

The method developed by Bartolommei and colleagues for measurement of ATPase activity has been used to measure the ATPase activity of Na/K-ATPase as well as ABC transporter ABCG2 expressed on the purified crude membrane (Bartolommei et al., 2013; Khunweeraphong et al., 2017). We have adopted this technique for our project due to its remarkable efficiency, sensitivity, and integrity.

**FIGURE 1:**
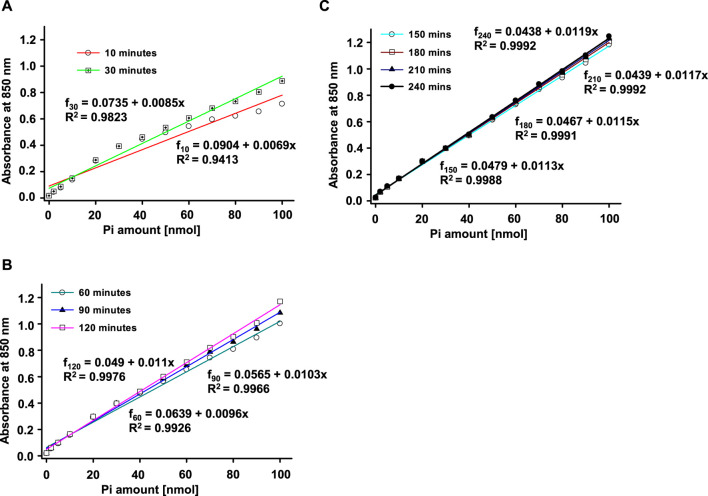
Phosphate standard curves used for the Antimony-phosphomolybdate assay. An example of a real time-dependent and concentration-dependent color development for Phosphate (P_i_) is presented in this figure. Color of the solution developed immediately **(A)** with absorbance measured at 850 nm. The color development stabilized at about 120 min **(B)** and remained stable with little to no further change after 120 min **(C)**. The solid lines represent linear fits (f = a + bx, where f is the reading/absorbance at 850 nm, and x is the amount of P_i_). Three individual experiments have been performed with similar results.

We freshly prepared 13 standard solutions of P_i_ in 100 µL final volume (including 0, 2, 5, 10, 20, 30, 40, 50, 60, 70, 80, 90,100 nmol) to obtain the calibration curve. Then, we added 900 µL of freshly mixed color solution and incubated the tubes at room temperature. Color started to develop immediately. To evaluate the color development, we observed the process at various intervals. As shown in Figure 1, we first detected the color absorbance at 10 min and proceeded with further detection until 240 min. The absorbance readings exhibited a continued increase until 90 min incubation, and then exhibited little change after 120 min. We fitted the data for these time courses with the Polynomial, Linear equation in SigmaPlot 12.3. The adjusted *R* ^2^ shifted from 0.9413 for 10 min duration to 0.9992 for 240 min duration. We have repeated the process for three different experiments in three individual days and the results are consistent. We then chose 120 min as the standard color development duration for this project.

### 3.2 Potential contamination of phosphate in ATP and PKA buffer solutions

**FIGURE 2:**
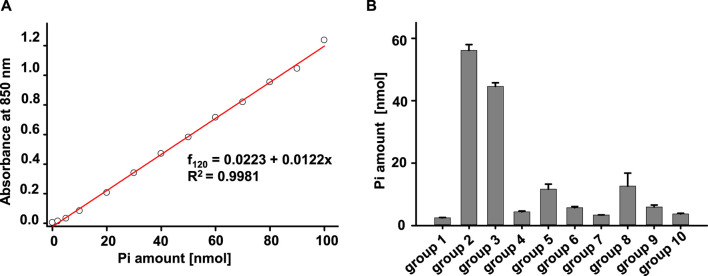
Evaluation of the effects of experiment solutions, ATP concentrations, and PKA concentrations using the Antimony-phosphomolybdate assay. **(A)** Standard curve of Phosphate measured with absorbance at 850 nm after a 2-h incubation. **(B)** Summary of amount of P_i_ measured in different solution combinations shown. The final volume for every group is 100 µL. The samples were incubated at room temperature for 1 h and then mixed with 900 µL color media for 2 h to allow for color development. The amount of P_i_ in each of the samples was detected by reading absorbance at 850 nm. The solutions mixed in each tube were: group 1, buffer +5 mM MgCl_2_. group 2, buffer +5 mM MgCl_2_ + 1 µL PKA (Promega). group 3: buffer +5 mM MgCl_2_ + 5 mM MgATP. group 4: buffer +5 mM MgCl_2_ + 1 µL PKA (1: 20 dilutions of original PKA in ddH_2_O) (Promega). group 5: buffer +5 mM MgCl_2_ + 1 mM MgATP. group 6: buffer +5 mM MgCl_2_ + 0.5 mM MgATP. group 7: buffer +5 mM MgCl_2_ + 0.1 mM MgATP. group 8: buffer +5 mM MgCl_2_ + 5 mM Na_2_ATP. group 9: buffer +5 mM MgCl_2_ + 1 mM Na_2_ATP. group 10: buffer +5 mM MgCl_2_ + 4 µL PKA (NEB). Three individual experiments for every group have been tested.

Our lab has been using MgATP (Sigma, A9187) and PKA (cAMP-dependent protein kinase, Catalytic subunit, Promega, Cat #V516A) to activate CFTR channels to study structure-function relationship in excised inside-out patches for decades. However, different groups studying CFTR have used diverse sources of ATP and PKA for their ATPase activity measurements. To help identify the experimental conditions leading to the most reproducible results for this study, we evaluated multiple different experiment combinations for their possible contributions of contaminants in the ATPase activity measurement. To identify conditions leading to the lowest apparent generation of P_i_ in the absence of ABC transporter protein, we tested ten different conditions including changes in concentration of MgCl_2_, MgATP, Na_2_ATP, and PKA. A representative calibration curve and summarized data are shown in Figures 2A,B, respectively. The data showed that the MgATP stock solution made with ddH_2_O contained phosphate. In addition, the storage buffer for PKA from Promega is 350 mM potassium phosphate, which matched our data showing that the amount of P_i_ in PKA from Promega is the highest among all the groups (Promega, V516A). Furthermore, buffer 2, the MgCl_2_ solution, the Na_2_ATP, as well as PKA from NEB contained minimal P_i_ and thus were used for the remainder of the study.

### 3.3 Endogenous proteins in the crude membranes of untransfected Sf9 cells exhibited ATPase activity

**FIGURE 3:**
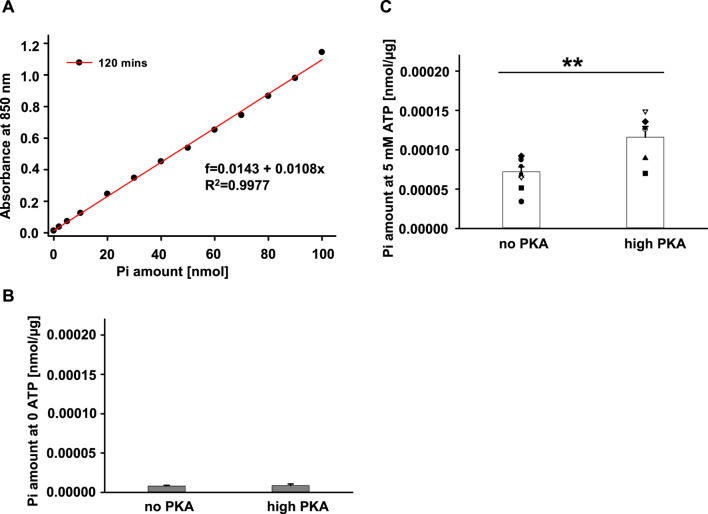
ATPase activity of endogenous protein expressed in the plasma membranes of untransfected Sf9 cells. ATPase activity of purified crude plasma membranes from native Sf9 cells was tested using Antimony-phosphomolybdate assay at 37°C for 20 min. **(A)** Representative standard curve of Phosphate measured with absorbance at 850 nm after a 2-h incubation. **(B)** Summary of amount of P_i_ released standardized by total amount of protein (µg). The total P_i_ amount measured in the Na_2_ATP- free condition together with the absence of PKA (no PKA) or with the presence of PKA (high PKA). **(C)** Summary of amount of P_i_ released standardized by total amount of protein (µg) used for the experiment. The P_i_ amount measured in the presence of 5 mM Na_2_ATP +5 mM MgCl_2_ together with the absence of PKA (no PKA) or with the presence of PKA (high PKA) (*n* = 5 for each), **p < 0.01.

A large population of proteins that carry ATPase activity are universally expressed in cells including Sf9 cells, such as ATP binding cassette family members, Na^+^/K^+^ ATPase, Ca^2+^-ATPase and more (Nitsche et al., 2018; Forzano et al., 2022). The endogenous ATPase activity in Sf9 cells could cause inaccuracy in measurements of ATPase activities of hABCC4, hCFTR, and Lp-CFTR. To estimate the possible effects of endogenous ATPase, we evaluated the endogenous ATPase activity of the crude membranes from untransfected Sf9 cells. A representative standard curve is shown in Figure 3A, and summary data are shown in Figures 3B,C, respectively. In the absence of Na_2_ATP (0 ATP), the crude membranes of untransfected Sf9 cells exhibited minimal ATPase activity with little P_i_ production regardless of the presence of PKA (Figure 3B). Not surprisingly, the membrane proteins produced significant amounts of P_i_ in the presence of 5 mM Na_2_ATP without PKA. Furthermore, the production of P_i_ was significantly higher in the presence of both ATP and PKA (Figure 3C). The data suggest that: 1) there is minimal contaminating ATP in the sample. 2) Some endogenous ATPase proteins were active in a PKA-independent manner. 3) PKA phosphorylated and activated endogenous proteins with ATPase activity and thus induced higher P_i_ production compared to ATP condition alone. In summary, the cell membranes from untransfected Sf9 cells contain endogenous proteins with ATPase activity, including proteins which exhibited PKA-dependent activity.

### 3.4 Semi-quantification of protein levels in crude plasma membrane

**FIGURE 4:**
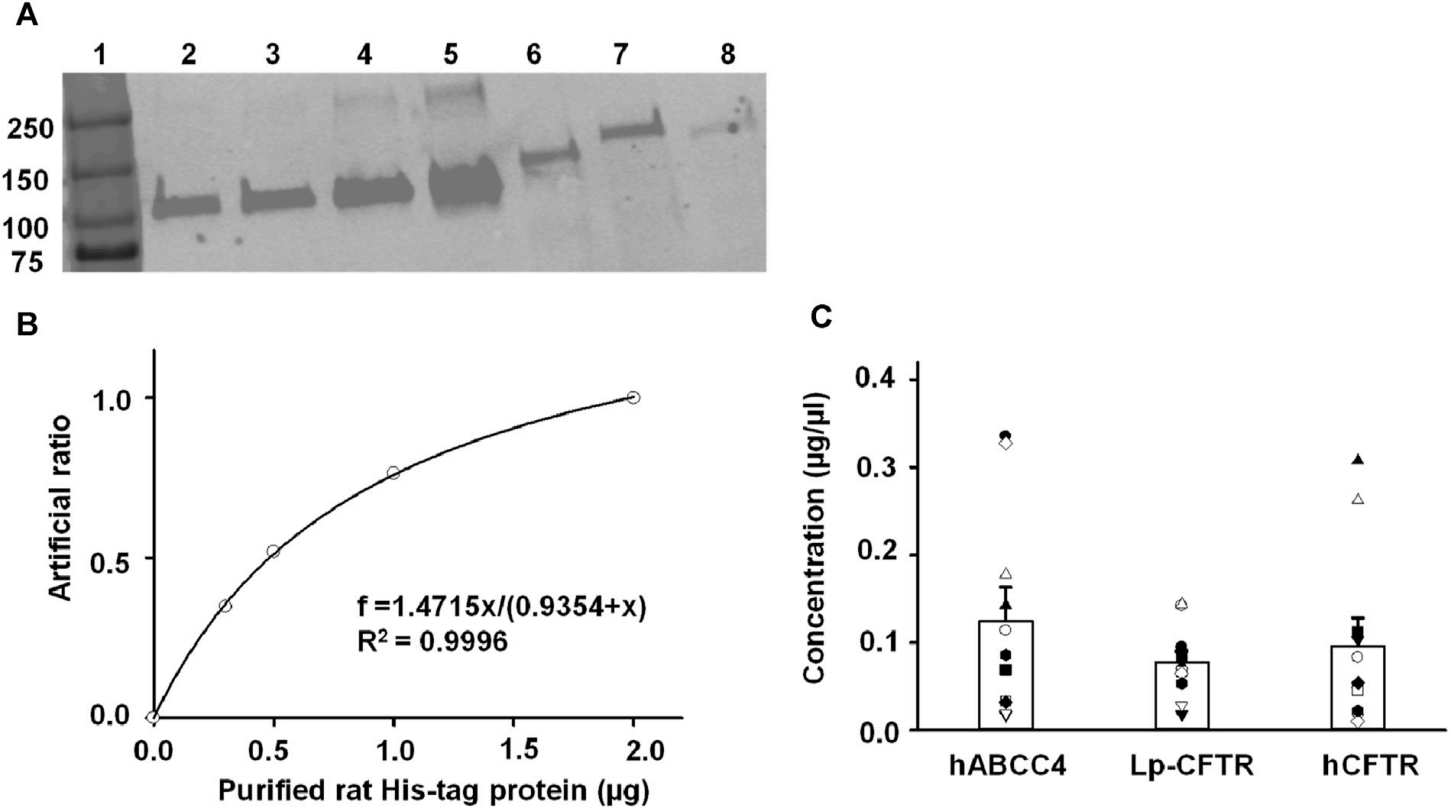
Semi-quantification of hABCC4, Lp-CFTR, and hCFTR protein levels in the crude plasma membranes purified from Sf9 cells using purified rat His-tag protein (PHRP) as a standard. **(A)** Representative Western blots for experiments containing serial dilution of PRHP and the three target proteins. Lanes 1 to 7 as follows: protein ladder, 200 ng PRHP, 300 ng PRHP, 500 ng PRHP, 1000 ng PRHP, plasma membranes expressing hABCC4, plasma membranes expressing Lp-CFTR, and plasma membranes expressing hCFTR. **(B)** Representative result of normalized Western blot fluorescent density (artificial ratio) plot to the amount of purified rat His-tag protein (µg). The solid line is the fit of the data with hyperbolic, Single Rectangular, 2 Parameter equation and the fit results are shown inside of the plot, with the adjusted *R* ^2^ = 0.9996. **(C)** Summary of the calculated protein concentration (µg/µL) for the three proteins tested. Each symbol represents an individual aliquot from the same membrane prep. There is no significant difference in the average concentration among proteins.

The results of a BCA assay only offer the total amount of protein in a sample. To evaluate the expression level of the three targeted protein, we adopted a semi-quantitative method utilizing immunoblotting to compare the ABC protein samples with a known loading protein. Figure 4 shows a representative western blot experiment with varying amounts of purified His-tag rat protein and three samples assessed with anti-His antibody. The band intensity was measured using ImageJ. The representative standard curve was fit with a hyperbolic, Single Rectangular, 2 Parameter equation in Sigmaplot 12.3 (Figure 4B). The concentration of each target protein was calculated based on their band intensities using the fit results from the standard curve. Summary data for the three target proteins are shown in Figure 4C where each symbol reflects analysis of a separate aliquot from the same membrane prep. Although the expression levels of each protein calculated by the method varied, no significant difference was observed among them. The results are not surprising because the overexpression of heterologous proteins could lead to cell death in the baculovirus/Sf9 cell system as well as in other systems. We chose to use the average protein concentration for each protein for the following experiments.

### 3.5 ATPase activity of hABCC4

**FIGURE 5:**
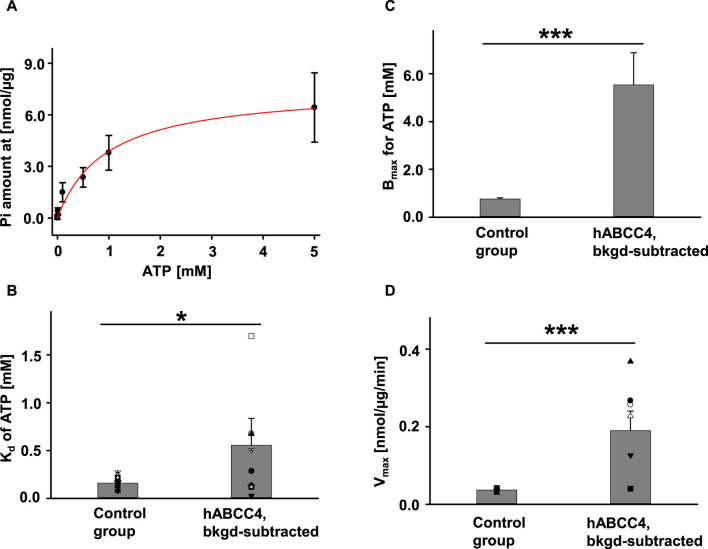
hABCC4 exhibited ATP concentration-dependent ATPase activity. **(A)** P_i_ released plotted against ATP concentration. The red solid line indicates the Michaelis-Menten fit. n = 5 for each ATP concentration. Summary of K_d_ and B_max_ are shown in **(B)** and **(C)**, respectively. **(D)** The maximum ATPase velocity (V_max_) was standardized by the total amount of protein for the control group and the background-subtracted hABCC4 group (hABCC4, bkgd-subtracted). *n* = 7 for each group. *p < 0.05; ***, *p* < 0.001 compared to control group in the individual panel.

Since activation of the hABCC4 protein does not require phosphorylation by PKA but only requires ATP, we measured the intrinsic ATPase activity of hABCC4 (i.e., in absence of substrate) in multiple ATP concentration conditions without PKA. We used membranes from untransfected Sf9 cells as control and loaded the same amount of total protein in every measurement. The ATPase activity of hABCC4 was ATP-concentration dependent (Figure 5A). We calculated total P_i_ released by hABCC4 by subtracting the amount of P_i_ released by the control group from that released by the hABCC4 group and divided by the estimated amount of hABCC4 protein (using the average concentration of hABCC4 calculated from Figure 4). We calculated the dissociation constant (K_d_) of ATP by fitting the data with Michaelis-Menten fits in Sigmaplot 12.3. K_d_s for the control group and background-subtracted hABCC4 are summarized in Figure 5B. K_d_ of hABCC4 for ATP was significantly higher than that of control group. We presented the estimated maximum ATP binding affinity (B_max_) for the control group and background-subtracted hABCC4 in Figure 5C. In addition, the B_max_ for hABCC4 was 5.53 ± 1.35 mM (*n* = 8), which was significantly higher than for the control group. The maximum ATPase activity (V_max_) for hABCC4 also was significantly higher than for the control group (Figure 5D).

### 3.6 ATPase activity of hCFTR and Lp-CFTR

**FIGURE 6:**
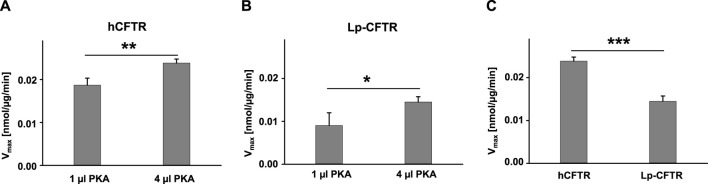
Lp-CFTR exhibited significant lower ATPase activity than hCFTR. **(A)** hCFTR exhibited higher ATPase activity in high PKA compared to low PKA conditions in the presence of 1 mM Na_2_ATP +5 mM MgCl_2_. **(B)** Lp-CFTR exhibited higher ATPase activity in high PKA compared to low PKA conditions in the presence of 1 mM Na_2_ATP +5 mM MgCl_2_. **(C)** hCFTR exhibited significantly higher ATPase activity compared to Lp-CFTR in the presence of high PKA. *n* = 4 for every group. *p < 0.05; **, *p* < 0.01; and ***, *p* < 0.001.

To make a comparison between hCFTR and Lp-CFTR, we measured their ATPase activity, subtracting activity of the endogenous proteins from untransfected Sf9 cell membranes as controls, in the same experimental conditions as shown in Figure 6. Both hCFTR and Lp-CFTR exhibited a PKA-concentration dependent ATPase activity (Figures 6A,B). Under the same experimental conditions, the ATPase activity of hCFTR was significantly higher compared to that of Lp-CFTR (Figure 6C). These data supported our previous findings that channel activity of hCFTR is driven by PKA-mediated phosphorylation and ATP hydrolysis, and is significantly stronger than that of Lp-CFTR (Cui et al., 2019).

### 3.7 Comparison of ATPase activity of hABCC4 and hCFTR

**FIGURE 7:**
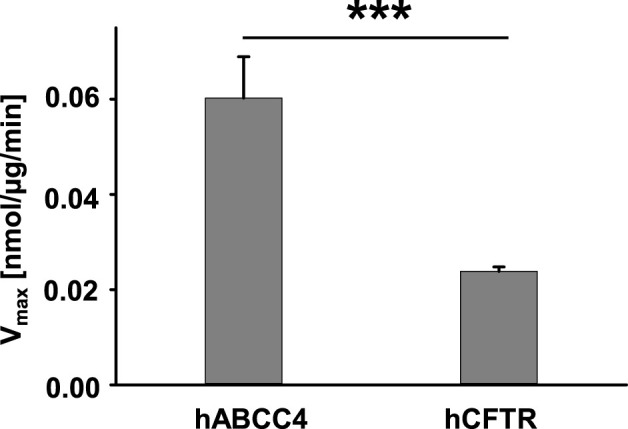
ATPase activity of hABCC4 is significantly higher than that of hCFTR in the presence of 1 mM Na_2_ATP +5 mM MgCl_2_. Membranes bearing hABCC4 were incubated in the presence of 1 mM Na_2_ATP +5 mM MgCl_2_ without PKA for 1 h at 30°C (*n* = 6). Membranes bearing hCFTR were incubated in the presence of 1 mM Na_2_ATP +5 mM MgCl_2_ and 4 µL PKA for 1 h at 30°C (*n* = 4). ***, *p* < 0.001 compared the two groups.

It is known that both hABCC4 and hCFTR contain two ATP binding sites, but only one site exhibits ATPase activity. As we have shown above, hABCC4 and hCFTR in Sf9 cell crude membranes exhibited similar protein expression levels (Figure 4). We compared the ATPase activity of hABCC4 and hCFTR evaluated with the same conditions of 1 mM Na_2_ATP and 5 mM MgCl_2_ at 30°C for 1 h incubation except with addition of PKA in the case of hCFTR (4 µL PKA from NEB = 10,000 units from NEB). The presence of PKA is a prerequisite for activating hCFTR (Liu et al., 2017; Zhang et al., 2017). The ATPase activity of hABCC4 is about twice that of hCFTR (Figure 7).

## 4 Discussion

In this study, we utilized the Baculovirus/Sf9 cell system and purified the crude membranes from Sf9 cells individually expressing hABCC4, Lp-CFTR, and hCFTR proteins. The crude membrane preps shared the same lipid composition and thus the proteins were embedded in the same lipid environment when their ATPase activities were evaluated. The data presented here suggest that the ATPase activity of hCFTR is significantly higher than that of Lp-CFTR. In addition, the intrinsic ATPase activity of hABCC4 tested in the absence of substrates is about twice of that of hCFTR albeit with slight difference in their experimental conditions (presence of PKA only in the latter).

As shown above, the concentrations of P_i_ (nmol) measured in the samples were normalized to the total protein (µg) used in the assay (nmol/µg). Although we measured all samples using a BCA assay to determine total protein concentrations for each, the abundance of the target proteins (hABCC4, Lp-CFTR, and hCFTR, each with a His-tag in the same epitope) in the individual samples is unknown. Thus, we adopted a semi-quantitative method to enable the comparison of ATPase activity in the samples. We used a known loading protein using the same anti-His antibody in each Western blotting experiment to generate a standard curve, which is the best approach available in this situation. Consequently, the rules for standard proteins in this assay are: 1) recognized by anti-His antibody; 2) the protein size is similar for the three target proteins (∼140 kDa); and 3) the standard protein is commercially available. We selected the best fit candidate, purified His-rat protein (PHRP), with molecular weight about 100 kDa for the semi-quantification experiment. We understand that several important factors could affect the results, including sample loading amount, sample fractionation, quantitative analyses requiring appropriate calibration curves, antibody specificity, and binding efficiency to different proteins, including the standard protein (Mollica et al., 2009; Murphy et al., 2013). Taking all of these things into consideration, we have assessed concentrations of the targeted proteins in the individual samples and their expression levels exhibited only very minor difference.

The ATPase activity of different proteins has been studied using a variety methods and techniques, including widely used NADH-coupled ATPase assay (Randak and Welsh, 2003; Liu et al., 2017; Zhang et al., 2017), a colorimetric assay (Villella et al., 2019; Bloch et al., 2023; Huang et al., 2023), radiation detection using conversion of radio-labeled ATP ([γ-^32^P]ATP) to [γ-^32^P]Pi (Rosenberg et al., 2004; Sauna et al., 2004; Ramjeesingh et al., 2008; Pasyk et al., 2009; Broadbent et al., 2015; Veit and Pomorski, 2023), a bioluminescence assay (Tai et al., 2011; Bloch et al., 2023), and the Antimony-phosphomolybdate assay (Bartolommei et al., 2013; Bartolommei and Tadini-Buoninsegni, 2016). We chose to use the Antimony-phosphomolybdate assay in this study because this method is highly sensitive, color-stable (non-fluorescent), and does not involve radiation.

Aside from the variety of ATPase activity measurement techniques, hCFTR has been studied in a wide range of other experimental conditions. For instance, 1) different cells that express hCFTR, such as HEK cells, Sf9 cells, or BHK cells; 2) different solutions, including ATP or PKA; and 3) crude membranes with CFTR expression, purified partial CFTR protein, or purified full-length CFTR protein. Consequently, the ATPase activity of hCFTR has ranged from less than 0.1 nmol/μg/min to over 130 nmol/μg/min. The K_d_ for ATP of hCFTR varied from about 50 µM to over 1 mM in these reported studies. Not only for hCFTR, but an analogous situation was also seen in the ATPase activity measurement of hABCC4. The ATPase activity of hABCC4 similarly has varied among a limited number of publications (Sauna et al., 2004; Huang et al., 2023). Furthermore, the variability of ATPase activities for hCFTR and hABCC4 could arise from many factors as addressed above; the lipid environment, and particularly the inclusion of detergent, is one of the key factors that significantly affects behavior of both proteins (Stauffer et al., 2017; Cottrill et al., 2020; Dunn et al., 2020; Cottrill et al., 2021; Cui et al., 2021; Sun et al., 2021; Bloch et al., 2023). In fact, membrane protein Cryo-EM structures solved in the presence of detergent exhibited significant differences compared to the structures embedded in lipids more closely mimicking their endogenous lipid environments (Chantemargue et al., 2018; Mio and Sato, 2018).

To the best of our knowledge, this is the first study directly comparing the ATPase activity of both hABCC4 and hCFTR that were expressed in the cell membrane with the same lipid environment. The results presented here were not derived from differences in the experimental conditions because: 1) we and other groups have studied hCFTR channels in excised inside-out patches which show that CFTR can be fully activated in the presence of ATP and PKA at room temperature within ∼5 min. Furthermore, hCFTR can be fully activated in whole cell recordings by forskolin or IBMX at room temperature in a similar time course (Wang et al., 2011; Cui et al., 2013; Cui et al., 2014; Wang et al., 2014; Wang et al., 2020). 2) hCFTR channel activity was only mildly increased when recorded at different temperatures from 24°C to 36°C (Aleksandrov and Riordan, 1998; Bagdany et al., 2017). 3) hABCC4 ATPase activity reached plateau in 20 min incubated at 37°C (Sauna et al., 2004; Huang et al., 2023), while hABCC4 ATPase activity reached similar level when incubated in 1 h at 30°C (Figure 7). Taken together, we conclude that the differences in experimental conditions in this study would not lead to a significant difference in the measured ATPase activity of hABCC4 and hCFTR. Phosphorylation of the R domain of hCFTR is required for its ATPase activity, while this does not happen in hABCC4. In addition, compared to the higher ATPase activity of ABCC4 in the presence of substrates, the intrinsic ATPase activity of ABCC4 measured here in the absence of substrate is lower (Wittgen et al., 2012).

In summary, we studied and compared the ATPase activity of hCFTR, hABCC4, and Lp-CFTR expressed in the same type of membranes with the same lipid environment. More studies are underway to identify the interaction sites between different lipids and the specific proteins, hCFTR, hABCC4, and Lp-CFTR, to fully understand the roles that lipids play in modulation of protein function. This study will guide further pharmacological therapy development and benefit patient populations with varied diseases (Moitra and Dean, 2011; Ford and Beis, 2019; Sumirtanurdin et al., 2019).

## Data Availability

The original contributions presented in the study are included in the article/Supplementary material, further inquiries can be directed to the corresponding author.
